# The Influence of L-Serine on Sperm Quality, Malondialdehyde, and Antioxidant Enzyme Activity After Cryopreservation in Bull Semen

**DOI:** 10.3390/ani16172809

**Published:** 2026-09-07

**Authors:** Ruthaiporn Ratchamak, Khomsan Buathalad, Maharach Matra, Eakapol Wangkahart, Wuttigrai Boonkum, Vibuntita Chankitisakul

**Affiliations:** 1 Division of Animal Science, Department of Agricultural Technology, Faculty of Technology, Mahasarakham University, Maha Sarakham 44150, Thailand; ruthaiporn.r@msu.ac.th (R.R.); komsan.bua@kkumail.com (K.B.); maharach.m@msu.ac.th (M.M.); 2 The Network Center for Animal Breeding and Omics Research, Khon Kaen University, Khon Kaen 40002, Thailand; wuttbo@kku.ac.th; 3 Division of Fisheries, Department of Agricultural Technology, Faculty of Technology, Mahasarakham University, Maha Sarakham 44150, Thailand; eakapol.w@msu.ac.th; 4 Department of Animal Science, Faculty of Agriculture, Khon Kaen University, Khon Kaen 40002, Thailand

**Keywords:** antioxidant, enzyme activity, L-serine, semen cryopreservation

## Abstract

Bull semen cryopreservation is essential for preserving valuable genetics and supporting cattle breeding programs; however, freeze–thaw stress compromises post-thaw sperm quality through oxidative damage and membrane injury. This study evaluated the effects of L-serine supplementation on post-thaw semen quality, susceptibility to induced lipid peroxidation, and antioxidant enzyme activity in bull semen. Semen collected from four Brahman bulls was cryopreserved in a Tris-based extender supplemented with 0, 2, 4, or 6 mM L-serine. Post-thaw total motility, progressive motility, viability, plasma membrane integrity, MDA concentration, and the activities of superoxide dismutase (SOD), glutathione peroxidase (GPx), and catalase (CAT) were assessed. Supplementation with 2 and 4 mM L-serine resulted in higher total motility and progressive motility, and greater plasma membrane integrity than the control and 6 mM treatments (*p* < 0.05), while viability was higher with 4 mM than with the control and 6 mM treatments (*p* < 0.05). MDA concentration following induced lipid peroxidation was lower in all L-serine-supplemented treatments than in the control (*p* < 0.05). SOD activity was higher with 2 mM than with the control and 6 mM treatments (*p* < 0.05), whereas GPx activity was highest with 2 mM, followed by 4 and 6 mM, with the lowest activity in the control (*p* < 0.05). CAT activity was unaffected by treatment (*p* = 0.1192). Overall, supplementation with 2–4 mM L-serine improved selected post-thaw sperm quality parameters and was associated with reduced susceptibility to induced lipid peroxidation and changes in apparent extracellular antioxidant enzyme activities. These findings support the potential of L-serine as a functional additive to improve bull semen cryopreservation.

## Simple Summary

Frozen bull semen is an essential tool in cattle breeding because it enables the long-term preservation and widespread use of superior genetics. However, the freezing and thawing process damages sperm cells through oxidative stress, reducing motility, viability, and membrane integrity. L-serine is a naturally occurring amino acid that supports cellular metabolism and antioxidant defense and may help protect sperm during cryopreservation. In this study, different concentrations of L-serine were added to a bull semen freezing extender to determine the optimal level for improving post-thaw semen quality. The results showed that 2–4 mM L-serine provided the most favorable responses in post-thaw sperm quality, whereas all L-serine-supplemented groups showed lower lipid peroxidation than the control. The antioxidant enzyme responses varied among concentrations, with the strongest GPx response observed at 2 mM. These findings indicate that L-serine may have potential as a functional additive for bull semen cryopreservation; however, fertility validation is required before its practical application in artificial insemination programs.

## 1. Introduction

Bull semen cryopreservation is an important reproductive technology that supports long-term preservation of valuable germplasm, facilitates use of genetically superior sires, and contributes to genetic improvement programs across production systems [1]. In tropical and subtropical regions, improving the quality of cryopreserved semen remains particularly relevant for sustaining cattle productivity and genetic resource management programs [2]. However, freeze–thaw stress exposes spermatozoa to cold shock, membrane phase transition, osmotic fluctuations, ice crystal injury, mitochondrial dysfunction, and oxidative imbalance, collectively reducing motility, viability, plasma membrane integrity, acrosome stability, and post-thaw functionality [3,4]. Among these mechanisms, oxidative stress is considered most critical, as excessive reactive oxygen species (ROS) attack membrane lipids, proteins, mitochondria, and DNA [5,6,7]. Malondialdehyde (MDA) is widely used as an indicator of oxidative damage, while superoxide dismutase (SOD), glutathione peroxidase (GPx), and catalase (CAT) reflect antioxidant capacity during cryopreservation [7,8,9].

Numerous studies have attempted to improve post-thaw sperm quality by supplementing freezing extenders with antioxidants, amino acids, or bioactive compounds. In bull semen, such supplementation has improved motility, viability, membrane integrity, and antioxidant capacity, though responses depend strongly on the type, concentration, and extender composition of the additive [10,11,12]. Importantly, beneficial effects do not increase linearly with dose; excessive supplementation may impair sperm quality and disrupt redox balance [13,14].

Among the various functional additives investigated for semen cryopreservation, amino acids have attracted considerable attention because of their roles in osmotic regulation, membrane maintenance, cellular metabolism, and antioxidant defense. Previous studies have demonstrated that cysteine and other amino acids can improve post-thaw sperm quality, although their effects depend on the amino acid type, concentration, species, and extender composition. Seminal plasma amino acid profiles have also been associated with bull sperm freezability, suggesting that amino acid metabolism may influence cryotolerance [6,15]. L-serine is of particular interest because it participates in glutathione synthesis, one-carbon metabolism, and phospholipid biosynthesis, processes that contribute to cellular redox homeostasis and membrane maintenance [16,17]. Previous studies have reported concentration-dependent effects of L-serine supplementation on preserved poultry and boar semen [18,19]. However, direct evidence regarding the effects of L-serine supplementation during bull semen cryopreservation remains limited, and its effective concentration range under bovine cryopreservation conditions has not been clearly defined.

The present study evaluated the effects of different levels of L-serine supplementation in freezing extender on post-thaw semen quality, lipid peroxidation, and antioxidant enzyme activity in bull semen. We hypothesized that L-serine would improve post-thaw sperm quality and influence lipid peroxidation and selected antioxidant enzyme activities in a concentration-related manner.

## 2. Materials and Methods

### 2.1. Animals and Management

The experiment was carried out at the MSU Farm of Mahasarakham University, Thailand, using four Brahman bulls aged 2 to 3 years. The bulls were in good physical condition and exhibited satisfactory sexual activity throughout the study. They were maintained individually in pens in an open-sided housing facility under approximately 12 h of natural daylight. Each bull received approximately 3 kg/day of a commercial concentrate containing 14% crude protein, with rice straw and drinking water available ad libitum.

All procedures involving animals were reviewed and approved by the Institutional Animal Care and Use Committee of Mahasarakham University (IACUC-MSU-104-106/2025).

### 2.2. Experimental Design

The study employed a split-ejaculate design. Six ejaculates were obtained from each of four bulls on separate collection occasions, providing 24 ejaculates in total. Immediately after collection and initial evaluation, each ejaculate was separated into four equal portions. The respective portions were then extended with Tris-based media containing 0, 2, 4, or 6 mM L-serine. The selected concentrations were based on previous findings in poultry and boar semen showing concentration-dependent responses to L-serine supplementation [18,19]. Thus, semen from every ejaculate was represented in all four treatment conditions, enabling treatment comparisons within the same ejaculate. For statistical purposes, an individual ejaculate subjected to a specific L-serine treatment constituted the experimental unit.

Post-thaw evaluation included total and progressive sperm motility, viability, and plasma membrane integrity. Lipid peroxidation was assessed from MDA concentration. These outcomes were evaluated using all 24 ejaculates obtained from four bulls. Antioxidant enzyme activities (SOD, GPx, and CAT) were evaluated in a subset comprising 12 ejaculates from three bulls (four ejaculates per bull), with each ejaculate represented in all four L-serine treatments. Measurements from technical replicates within each ejaculate–treatment aliquot were averaged before statistical analysis.

### 2.3. Chemicals and Media Preparation

Unless specified otherwise, analytical-grade chemicals were purchased from Sigma-Aldrich (St. Louis, MO, USA). Reagents were prepared aseptically, and individual components were measured using an analytical balance with a readability of 0.1 mg. Before use, the extender was passed through a 0.22 µm syringe filter and kept at 4 °C overnight.

The Tris–egg yolk extender was formulated in 100 mL of ultrapure water and contained 20% (*v*/*v*) egg yolk, 1.675 g citric acid monohydrate, 1.250 g fructose, 3.028 g Tris base, 0.100 g streptomycin, and 0.06 g penicillin. The prepared extender was subsequently aliquoted and supplemented with L-serine to obtain final treatment concentrations of 0, 2, 4, or 6 mM. For each L-serine level, the extender was prepared as two separate fractions for sequential semen dilution. The first fraction contained no glycerol and was used during initial dilution and cooling, whereas the second fraction contained 14% (*v*/*v*) glycerol. After cooling, the glycerol-containing fraction was combined with the semen at an equal volume (1:1, *v*/*v*), yielding a final glycerol concentration of 7% (*v*/*v*) in every treatment [13,14].

### 2.4. Semen Collection

Semen was obtained once per week from each bull using an electroejaculator (Pulsator IV; Lane Manufacturing, Denver, CO, USA). This collection technique was used because it represented the routine procedure for breeding bulls at the experimental farm, and the study bulls had not previously been trained for collection with an artificial vagina. Electroejaculation has also been applied previously for the collection and subsequent cryopreservation of bovine semen [20,21,22]. Although the collection technique can affect ejaculate characteristics, including volume, sperm concentration, and seminal plasma composition, its potential influence on treatment comparisons was minimized by the split-ejaculate design, in which all treatment aliquots originated from the same ejaculate.

Prior to semen collection, feces were manually evacuated from the rectum. A lubricated rectal probe was then positioned with its electrodes facing ventrally. Using the automatic operating mode, electrical stimulation was delivered for a maximum of three cycles with gradually increasing intensity. Each stimulus lasted 2–3 s and was separated by a 2 s interval. Stimulation was stopped as soon as ejaculation occurred, in accordance with the procedure described by Montoya-Monsalve et al. [23]. Semen was collected directly into a sterile graduated 15 mL centrifuge tube, and ejaculate volume was recorded immediately from the tube graduations.

Following collection, samples were maintained at 37 °C during transport to the laboratory and evaluated without delay. Total sperm motility was determined within 30 min using phase-contrast microscopy at 400× magnification while the samples were maintained at 37 °C on a temperature-controlled warming plate. Viability was assessed by eosin–nigrosin staining, whereas sperm concentration was determined with a hemocytometer after a 1:200 dilution in 4% sodium chloride solution for sperm immobilization. Only ejaculates with total motility ≥ 85% and sperm concentration ≥ 400 × 10^6^ sperm/mL were included in the cryopreservation experiment, according to the criteria described by Authaida et al. [13]. Overall, 24 ejaculates, representing six collections from each of the four bulls, were included in the study.

### 2.5. Semen Cryopreservation and Thawing

Semen cryopreservation was performed using a protocol adapted from Authaida et al. [13]. Following initial semen evaluation, each ejaculate was separated into four equal portions corresponding to the four L-serine treatments (0, 2, 4, and 6 mM). At 30 °C, each portion was first extended with the appropriate glycerol-free Tris-based extender to obtain a sperm concentration of 80 × 10^6^ sperm/mL. The diluted semen was then cooled progressively from 37 to 5 °C over a 2 h period.

Once the samples reached 5 °C, an equal volume of the corresponding glycerol-containing extender (14% glycerol, *v*/*v*) containing the same concentration of L-serine was added at a 1:1 (*v*/*v*) ratio. This second dilution resulted in final concentrations of 7% (*v*/*v*) glycerol and 40 × 10^6^ sperm/mL in all treatment groups [13,14]. The diluted samples were maintained at 5 °C for a further 3 h to allow equilibration, then filled into 0.5 mL straws.

For freezing, the straws were positioned horizontally 4 cm above the surface of liquid nitrogen in a Styrofoam container (25 × 35 × 30 cm) for 15 min. They were then immersed directly in liquid nitrogen (−196 °C) for storage. Prior to post-thaw evaluation, individual straws were warmed in a 37 °C water bath for 30 s.

### 2.6. Post-Thaw Sperm Quality Assessment

#### 2.6.1. Total and Progressive Sperm Motility

Post-thaw total and progressive sperm motility were determined using a computer-assisted sperm analysis (CASA) system (HTM-IVOS, Hamilton Thorne Biosciences, Beverly, MA, USA; software version 10). A pre-warmed 2X-CEL chamber slide (Hamilton Thorne Inc., Beverly, MA, USA) with a depth of 20 µm was loaded with 5 µL of semen sample and maintained at 37 °C. Images were acquired at 60 frames/s for 30 frames per field. Spermatozoa were classified as translationally motile when motility was defined as average path velocity (VAP) > 5 µm/s and straight line velocity (VSL) > 3 µm/s, and progressive motility was defined as VAP ≥ 20 µm/s and straightness (STR) ≥ 80%. For each assessment, 5 µL of thawed semen was introduced into an analysis chamber pre-warmed to 37 °C. At least 500 spermatozoa from a minimum of five microscopic fields were examined for each sample. The CASA assessment was restricted to total and progressive motility, and sperm kinematic variables were not included in the present study. Total motility represented the proportion of spermatozoa showing detectable movement, whereas progressive motility represented those exhibiting forward progression.

#### 2.6.2. Sperm Viability

Post-thaw sperm viability was evaluated by eosin–nigrosin staining. A 15 µL semen aliquot was combined with 30 µL of eosin–nigrosin stain on a glass slide and spread to form a thin smear. After complete air-drying, the preparations were examined under oil immersion at 1000× magnification using a light microscope. At least 300 spermatozoa were counted from each sample. Cells that excluded the eosin stain were classified as viable, whereas spermatozoa showing pink eosin staining were considered non-viable. Viability was calculated as the percentage of viable spermatozoa among the total number of cells examined.

#### 2.6.3. Hypo-Osmotic Swelling Test (HOST)

Functional integrity of the sperm plasma membrane was examined using the hypo-osmotic swelling test (HOST) [24]. The hypo-osmotic medium was prepared by dissolving 0.735 g sodium citrate and 1.351 g fructose in 100 mL of distilled water, producing an osmolarity of 190 mOsmol/kg as described by Elkhawagah et al. [24]. For the assay, 50 µL of thawed semen from each treatment was combined with 500 µL of HOST solution and incubated for 40 min at 37 °C. Samples were subsequently examined under an Olympus microscope (Japan) at 400× magnification. A minimum of 200 spermatozoa was counted per sample, and sperm showing characteristic swelling and coiling of the tail were considered to have functionally intact plasma membranes.

### 2.7. Lipid Peroxidation

Lipid peroxidation was quantified from malondialdehyde (MDA) concentration using the thiobarbituric acid (TBA) reaction according to Ratchamak et al. [25]. Semen samples were first exposed to 0.2 mM ferrous sulfate (FeSO_4_) and 1 mM ascorbic acid for 60 min at 37 °C. Thereafter, 15% (*w*/*v*) trichloroacetic acid and 0.375% (*w*/*v*) thiobarbituric acid were added to the reaction mixture, which was heated in a boiling water bath for 10 min. The reaction was stopped by cooling the samples to 4 °C. Following centrifugation at 800× *g* for 10 min, the absorbance of the resulting supernatant was determined at 532 nm using a UV–Vis spectrophotometer (Specord 250 Plus, Analytik Jena, Jena, Germany). MDA concentration was reported as µmol/mL. Because samples were exposed to FeSO_4_ and ascorbic acid before the TBA reaction, the resulting MDA values represent MDA formation under induced lipid peroxidation conditions and therefore reflect the susceptibility of post-thaw semen to peroxidative challenge rather than basal post-thaw MDA concentration.

### 2.8. Antioxidant Enzyme Activity

Antioxidant enzyme assays were performed on a subset of 12 ejaculates obtained from three bulls (four ejaculates per bull), representing the samples allocated for biochemical enzyme analysis. Each ejaculate included in this subset contributed samples to all four L-serine treatments. For antioxidant enzyme assays, semen samples were centrifuged at 4000× *g* for 10 min at room temperature to obtain the extracellular supernatant fraction of the extended semen. This fraction contained extracellular seminal constituents together with components of the semen extender and was therefore not considered equivalent to native seminal plasma. The separated supernatant was stored at −20 °C until analysis. Measurements were performed in 96-well microplates using a VersaMax™ microplate reader (Molecular Devices, San Jose, CA, USA). Unless otherwise specified, enzyme activities were expressed as units per milliliter (U/mL) [19].

#### 2.8.1. Glutathione Peroxidase (GPx)

Glutathione peroxidase activity was determined using a coupled enzyme assay. Briefly, 20 μL of extended-semen supernatant was combined with 20 μL of 0.1% Triton X-100 prepared in phosphate-buffered saline (PBS; pH 7.4). The mixture was supplemented with 20 μL of a reagent containing 24 μM reduced glutathione (GSH), 4.8 μM nicotinamide adenine dinucleotide phosphate (NADPH), and 12 U glutathione reductase, followed by the addition of 35 μL of 1% H_2_O_2_. After incubation for 3 min at room temperature, absorbance at 450 nm was measured at 30 and 90 s. GPx activity was expressed in U/mL and calculated as OD_450_/0.00622.

#### 2.8.2. Superoxide Dismutase (SOD)

Superoxide dismutase activity was evaluated using the epinephrine autooxidation assay. Extended-semen supernatant (20 μL) was mixed with 200 μL of sodium carbonate buffer (pH 10.2) containing 30 mM epinephrine prepared in HCl. Absorbance at 490 nm was recorded at 30 and 90 s. SOD activity was calculated as [100 − (ΔODsample/ΔODcontrol × 100)] × 3.75, where ΔODsample represents the change in absorbance of the seminal plasma sample and ΔODcontrol represents the corresponding change in the blank control. Results were expressed as U/mL.

#### 2.8.3. Catalase (CAT)

Catalase activity was assessed from the decomposition of H_2_O_2_. A 10 μL aliquot of the extracellular supernatant fraction of post-thaw extended semen was mixed with 50 μL of 10% H_2_O_2_ and 50 μL of PBS (pH 7.4). Absorbance was measured at 240 nm at 20 and 80 s. CAT activity was expressed as U/mL and calculated as (OD1 − OD2)/0.0008, where OD1 and OD2 are the absorbance values measured at 20 and 80 s, respectively.

### 2.9. Statistical Analysis

All statistical analyses were conducted using SAS version 9.4 (SAS Institute Inc., Cary, NC, USA). Each measured outcome was analyzed independently with a linear mixed-effects model. L-serine concentration was specified as the fixed effect, while bull and ejaculate nested within bull were incorporated as random effects to account for variation among animals and repeated ejaculates within individual bulls. The overall effect of L-serine concentration was assessed using an F-test, whereas the covariance parameters associated with the random effects were evaluated using Wald Z-tests.

When a significant overall treatment effect was detected, least-squares means were compared using Tukey-adjusted pairwise tests. SOD, GPx, and CAT activities were analyzed using the available subset of 12 ejaculates from three bulls (four ejaculates per bull), with all four L-serine treatments represented in each ejaculate; consequently, the degrees of freedom in the denominator for these variables differed from those of the other response traits. Percentage variables, including total motility, progressive motility, viability, and plasma membrane integrity, were analyzed on their original scale without transformation. Model assumptions were evaluated by assessing residual normality using the Shapiro–Wilk test and homogeneity of variance using Levene’s test. Because the residual diagnostics did not indicate substantial departures from these assumptions, no transformation of the percentage variables was considered necessary. A homogeneous residual variance structure was assumed across L-serine treatment groups, with no treatment-specific heterogeneity in variance. Statistical significance was declared at *p* < 0.05.

## 3. Results

### 3.1. Fresh Semen Quality

Fresh semen quality parameters before cryopreservation are presented in Table 1. All ejaculates met the minimum inclusion criteria for total motility and sperm concentration and also exhibited high initial viability and were therefore considered suitable for cryopreservation.

### 3.2. Effect of L-Serine on Post-Thaw Semen Quality

L-serine supplementation affected all measured sperm quality parameters (*p* < 0.0001; Table 2). Supplementation with 2 and 4 mM L-serine resulted in higher total motility, progressive motility, and plasma membrane integrity than the control and 6 mM groups (*p* < 0.05), with no differences between the 2 and 4 mM treatments for these parameters (*p* > 0.05). Sperm viability was higher in the 4 mM treatment than in the control and 6 mM treatments (*p* < 0.05), whereas the 2 mM treatment did not differ from any other treatments (*p* > 0.05). The 6 mM treatment did not differ from the control in any of the measured post-thaw sperm quality parameters (*p* > 0.05).

### 3.3. Effect of L-Serine on MDA Concentration and Antioxidant Enzyme Activity

L-serine concentration affected MDA formation following induced lipid peroxidation, SOD activity, and GPx activity (*p* < 0.01), whereas CAT activity was unaffected (*p* = 0.1192; Table 3). MDA concentration measured after induced lipid peroxidation was lower in all L-serine-supplemented treatments (2, 4, and 6 mM) than in the control (*p* < 0.05), with no differences among the three supplemented treatments (*p* > 0.05). SOD activity was higher in the 2 mM treatment than in the control and 6 mM treatments (*p* < 0.05), whereas the 4 mM treatment did not differ from any other treatment (*p* > 0.05). GPx activity was highest in the 2 mM treatment, followed by the 4 and 6 mM treatments, whereas the control showed the lowest activity (*p* < 0.05); the 4 and 6 mM treatments did not differ from each other (*p* > 0.05). CAT activity did not differ among treatments (*p* = 0.1192).

## 4. Discussion

The present study demonstrated that L-serine supplementation influenced post-thaw sperm quality, lipid peroxidation, and selected antioxidant enzyme activities. The most favorable sperm quality responses were observed at 2–4 mM, whereas all L-serine-supplemented treatments showed lower MDA concentrations than the control. Antioxidant enzyme responses differed among concentrations, with 2 mM producing the greatest GPx activity and a higher SOD activity than the control and 6 mM treatments. In contrast, 6 mM did not improve post-thaw motility, viability, or plasma membrane integrity relative to the control. These findings are consistent with previous reports indicating that cryopreservation-induced oxidative stress is a major contributor to sperm dysfunction through membrane destabilization, mitochondrial impairment, and excessive production of reactive oxygen species [5,6,7,26]. However, because oxidative stress was evaluated using only MDA and selected antioxidant enzymes, the present results should be interpreted as evidence of changes in lipid peroxidation and antioxidant enzyme activity rather than as proof that oxidative stress was prevented. Collectively, the findings suggest that appropriately optimized L-serine supplementation may help preserve selected aspects of post-thaw sperm function during bull semen cryopreservation.

The higher total and progressive motility and plasma membrane integrity observed with 2 and 4 mM L-serine, together with the higher viability observed at 4 mM, suggest that L-serine supplementation within this concentration range may help preserve selected aspects of sperm function during cryopreservation. Sperm motility depends on the maintenance of plasma membrane integrity and cellular energy metabolism, both of which are particularly susceptible to cryoinjury. Therefore, the improved sperm quality observed in the present study is consistent with enhanced preservation of sperm structural and functional integrity during the freeze–thaw process. Similar improvements in post-thaw sperm quality have been reported following supplementation of semen extenders with antioxidant compounds in bull semen [7,12,27,28,29,30,31,32]. In addition to its potential role in antioxidant metabolism, L-serine serves as a precursor for phosphatidylserine and sphingolipid biosynthesis, both of which are important structural components of biological membranes [17,33]. Although membrane lipid composition was not evaluated in the present study, these metabolic functions provide a biologically plausible explanation for the improved plasma membrane integrity and post-thaw sperm quality observed following L-serine supplementation.

MDA concentration following induced lipid peroxidation was lower in all L-serine-supplemented treatments than in the control (*p* < 0.05), with no differences among the 2, 4, and 6 mM treatments (*p* > 0.05). Because sperm plasma membranes are highly enriched with polyunsaturated fatty acids, they are particularly vulnerable to oxidative damage induced by freeze–thaw stress, which can compromise membrane function and ultimately impair sperm motility and viability [34,35,36]. Interestingly, although the 6 mM treatment showed a lower MDA concentration than the control, it did not improve post-thaw motility, viability, or plasma membrane integrity. This finding suggests that reduced lipid peroxidation alone may not fully explain the effects of L-serine on post-thaw sperm function. Therefore, the present findings support an association between L-serine supplementation and reduced susceptibility to induced lipid peroxidation across the concentrations evaluated but do not indicate a concentration-specific advantage of 4 mM for this endpoint. Similar reductions in lipid peroxidation accompanied by improvements in post-thaw sperm quality have also been reported following antioxidant supplementation in bull semen [7,12,13,14].

The antioxidant enzyme responses varied among L-serine concentrations. SOD activity was higher in the 2 mM treatment than in the control and 6 mM treatments (*p* < 0.05), whereas the 4 mM treatment showed an intermediate response and did not differ from the other treatments (*p* > 0.05). GPx activity showed a clear treatment pattern, with the highest activity at 2 mM, intermediate activity at 4 and 6 mM, and the lowest in the control (*p* < 0.05). SOD and GPx contribute to antioxidant defense by converting superoxide radicals and hydrogen peroxide into less reactive molecules, thereby helping to limit oxidative damage and lipid peroxidation [26,34,37,38]. Similar changes in antioxidant enzyme activities following antioxidant supplementation have been reported in frozen–thawed bull and buffalo semen [7,26,27,28,30]. Collectively, these findings indicate that L-serine supplementation was associated with changes in selected antioxidant enzyme activities in post-thaw extended semen. However, the different response patterns among SOD, GPx, and MDA do not establish distinct concentration-specific protective mechanisms.

CAT and GPx contribute to the removal of hydrogen peroxide through distinct enzymatic mechanisms. CAT converts hydrogen peroxide directly into water and oxygen, whereas GPx uses reduced glutathione to reduce hydrogen peroxide and lipid hydroperoxides. Previous studies have reported limited intrinsic antioxidant enzyme activity in bovine spermatozoa after separation from seminal plasma [39]. However, the antioxidant enzyme activities measured in the present study were determined in the extracellular supernatant fraction of post-thaw-extended semen rather than in spermatozoa. Therefore, these measurements should be interpreted as apparent extracellular antioxidant enzyme activities under the present cryopreservation conditions and may include contributions from both extracellular seminal constituents and components of the Tris–egg yolk extender. Accordingly, they should not be considered direct measures of intrinsic sperm antioxidant capacity. Previous work has also indicated that the extracellular antioxidant environment of bovine semen is associated with sperm susceptibility to oxidative damage during cryopreservation [40]. In the present study, GPx activity was highest in the 2 mM treatment, whereas the 4 and 6 mM treatments showed intermediate activities that remained higher than the control (*p* < 0.05). In contrast, CAT activity did not differ among treatments (*p* = 0.1192). These findings indicate treatment-associated differences in extracellular GPx activity but should not be interpreted as evidence of intracellular enzyme activation or dominance of a specific antioxidant pathway.

The concentration-related responses observed in the present study should be interpreted in the context of species-specific sperm biology and semen preservation systems. Supplementation with 2–4 mM L-serine produced the most favorable post-thaw sperm quality responses in bull semen, which is broadly consistent with effective concentrations reported in cryopreserved chicken semen and liquid-stored rooster semen [18,41]. In contrast, a lower optimal concentration has been reported for boar semen cryopreservation, whereas higher concentrations impaired post-thaw sperm function [19]. These findings indicate that the optimal concentration of L-serine cannot be generalized across species or preservation systems. Such variation is likely attributable to differences in sperm membrane composition, membrane lipid organization, osmotic tolerance, and endogenous antioxidant capacity, all of which influence susceptibility to cryoinjury and oxidative stress during preservation [4,42,43].

The mechanisms underlying the observed responses were not directly investigated. L-serine participates in metabolic pathways related to cellular redox regulation and membrane biosynthesis [16,17,44,45], which may help explain its effects during cryopreservation. However, glutathione status, one-carbon metabolism, phospholipid synthesis, and sphingolipid metabolism were not evaluated in the present study. Therefore, these pathways should be regarded only as hypotheses, and the current findings should be interpreted primarily based on measured sperm quality, MDA concentration, and antioxidant enzyme activities.

The lack of improvement in post-thaw sperm quality at 6 mM, despite lower MDA concentration and higher GPx activity than the control, further indicates that changes in individual oxidative indicators do not necessarily translate into improved sperm function. This pattern also suggests that increasing L-serine concentration does not produce proportional functional benefits during bull semen cryopreservation. Similar non-linear responses have been reported for several amino acid and antioxidant additives used in semen preservation. For example, L-cysteine and related compounds exhibit dose-dependent effects on frozen bull semen quality, whereas higher concentrations of L-serine impair post-thaw boar sperm function [15,19]. Likewise, GSH-selenium nanoparticles, Moringa oleifera leaf extract, and Kaempferia parviflora improved post-thaw sperm quality only within an optimal concentration range, while excessive supplementation diminished these benefits [7,13,14].

The mechanisms underlying reduced efficacy at higher concentrations remain unclear but may involve disruption of osmotic balance, metabolic homeostasis, or physiological redox regulation. Because sperm function depends on a tightly regulated balance between ROS production and antioxidant defenses, excessive antioxidant supplementation does not necessarily provide greater cryoprotection. Collectively, these findings indicate that responses to L-serine are concentration-related and endpoint-dependent rather than proportional to concentration, highlighting the importance of optimizing supplementation based on the specific functional outcome, species, and semen preservation protocol.

### Study Limitations

Several limitations of the present study should be considered. First, the experiment included only four Brahman bulls, although six ejaculates per bull and a split-ejaculate design were used. Therefore, the findings may not fully represent the biological variability among wider populations of Brahman bulls, other cattle breeds, or other species. Nevertheless, compared with the control, the estimated treatment differences included a 19.55-percentage-point increase in total motility with 2 mM L-serine (95% CI: 14.98 to 24.12), a 6.31-percentage-point increase in viability with 4 mM L-serine (95% CI: 3.91 to 8.71), and a 0.21 µmol/mL reduction in MDA formation following induced lipid peroxidation with 4 mM L-serine (95% CI: 0.18 to 0.24). These estimates and their confidence intervals should be interpreted with caution because the study included a limited number of bulls and therefore require confirmation in larger and more diverse populations. In addition, antioxidant enzyme activities were evaluated in a subset comprising only 12 ejaculates from three bulls. Therefore, the findings for SOD, GPx, and CAT are based on a smaller number of animals and ejaculates than the sperm quality and MDA outcomes and may have more limited generalizability. These enzyme-related findings should therefore be confirmed in larger studies involving more bulls and ejaculates. Second, sperm viability, concentration, and plasma membrane integrity were evaluated using conventional microscopy-based methods, which may be subject to observer-related variation despite standardized procedures and predetermined sperm counts. More advanced assessments, including fluorescent membrane and acrosome integrity assays, mitochondrial activity, sperm DNA integrity, and detailed CASA-derived kinematic parameters, were not performed.

Third, susceptibility to lipid peroxidation and selected extracellular antioxidant enzyme activities were evaluated using MDA formation following an induced lipid-peroxidation challenge and the activities of SOD, GPx, and CAT, respectively. These measurements represent selected indicators of oxidative processes and antioxidant defense and do not constitute a comprehensive assessment of oxidative stress. Direct ROS production, glutathione status, total antioxidant capacity, and oxidative damage to sperm proteins or DNA were not evaluated. In addition, antioxidant enzyme activities were measured in the extracellular supernatant of post-thaw extended semen rather than within spermatozoa. Because this fraction contained both extracellular seminal constituents and components of the Tris–egg yolk extender, the measured enzyme activities should not be interpreted as direct indicators of intrinsic sperm antioxidant capacity.

Finally, no assessments of in vivo fertility, in vitro fertilization, or sperm–oocyte interaction were conducted. Therefore, the observed improvements in post-thaw sperm quality and reduced susceptibility to induced lipid peroxidation cannot be assumed to result in improved fertilizing capacity. Evaluating the effects of L-serine supplementation on sperm–oocyte binding, IVF outcomes, conception rate, and pregnancy establishment should be considered a priority before its practical application in artificial insemination programs. Further studies involving larger and more diverse bull populations, comprehensive functional and oxidative assessments, and fertility endpoints are therefore required.

## 5. Conclusions

This study demonstrated that L-serine supplementation influenced post-thaw sperm quality, lipid peroxidation, and selected antioxidant enzyme activities in cryopreserved bull semen. Supplementation at 2–4 mM produced the most favorable post-thaw sperm quality responses, whereas all L-serine-supplemented treatments reduced MDA concentration relative to the control. Antioxidant enzyme responses varied among concentrations, with the greatest GPx activity observed at 2 mM. These findings indicate that L-serine may have potential as a functional additive for bull semen cryopreservation; however, validation in larger bull populations and evaluation of functional fertility outcomes are required before practical application in artificial insemination programs.

## Figures and Tables

**Table 1:** Fresh semen quality parameters of Brahman bulls (*n* = 24 ejaculates from four bulls).

Parameter	Mean ± SE
Volume (mL)	10.00 ± 1.10
Concentration (×10^6^ sperm/mL)	762.25 ± 25.10
Viability (%)	91.35 ± 1.48
Total Motility (%)	90.15 ± 1.10

**Table 2:** Effects of L-serine supplementation on post-thaw semen quality in cryopreserved bull semen.

L-Serine Levels	Total Motility (%)	Progressive Motility (%)	Viability (%)	Plasma Membrane Integrity (%)
Control	37.90 ± 1.09 ^b^	24.70 ± 0.74 ^b^	48.44 ± 1.90 ^b^	40.50 ± 2.50 ^b^
L-serine 2 mM	57.45 ± 1.74 ^a^	33.40 ± 0.57 ^a^	50.88 ± 1.97 ^ab^	47.88 ± 2.83 ^a^
L-serine 4 mM	54.07 ± 3.75 ^a^	33.42 ± 1.62 ^a^	54.75 ± 2.29 ^a^	44.56 ± 2.46 ^a^
L-serine 6 mM	38.16 ± 1.85 ^b^	23.24 ± 0.90 ^b^	47.88 ± 1.99 ^b^	38.81 ± 3.08 ^b^
*F*-value	40.71	29.53	13.45	68.72
df	3, 69	3, 69	3, 69	3, 69
*p*-values_(Treatment)_	<0.0001	<0.0001	<0.0001	<0.0001
*p*-values_(Bull)_	0.0829	0.4359	0.1465	0.0994
*p*-values_[Ejaculate(Bull)]_	0.1330	0.3382	0.0027	0.0016

Values are presented as least-squares means ± SE obtained from the linear mixed-effects model. Within each column, means with different superscript letters differ significantly (*p* < 0.05) based on Tukey-adjusted pairwise comparisons of least-squares means from the same model.

**Table 3:** Effects of L-serine supplementation on MDA concentration following induced lipid peroxidation and antioxidant enzyme activity in cryopreserved bull semen.

L-Serine Levels	MDA (µmol/mL)	Antioxidant Enzyme Activity (U/mL)		
		SOD	GPx	CAT
Control	0.62 ± 0.03 ^b^	242.87 ± 1.34 ^b^	21.82 ± 2.06 ^c^	26.48 ± 0.45
L-serine 2 mM	0.46 ± 0.02 ^a^	248.84 ± 0.50 ^a^	26.07 ± 2.37 ^a^	25.79 ± 0.07
L-serine 4 mM	0.41 ± 0.02 ^a^	246.51 ± 1.42 ^ab^	23.84 ± 2.39 ^b^	26.50 ± 0.31
L-serine 6 mM	0.48 ± 0.02 ^a^	243.40 ± 0.65 ^b^	24.07 ± 1.86 ^b^	26.12 ± 0.16
*F*-value	69.11	7.85	20.73	2.10
df	3, 69	3, 33	3, 33	3, 33
*p*-values_(Treatment)_	<0.0001	0.0004	<0.0001	0.1192
*p*-values_(Bull)_	0.0750	0.4852	0.2174	0.2135
*p*-values_[Ejaculate(Bull)]_	0.0155	0.2505	0.0181	0.1988

Values are presented as least-squares means ± SE obtained from the linear mixed-effects model. Within each column, means with different superscript letters differ significantly (*p* < 0.05) based on Tukey-adjusted pairwise comparisons of least-squares means from the same model.

## Data Availability

The data are available upon request from the corresponding author.
